# Two Photon lithography additive manufacturing: Video dataset of parameter sweep of light dosages, photo-curable resins, and structures

**DOI:** 10.1016/j.dib.2020.106119

**Published:** 2020-08-03

**Authors:** Xian Yeow Lee, Sourabh K. Saha, Soumik Sarkar, Brian Giera

**Affiliations:** a Department of Mechanical Engineering, Iowa State University, United States; b G.W. Woodruff School of Mechanical Engineering, Georgia Institute of Technology, United States; c Lawrence Livermore National Laboratory, United States

**Keywords:** Additive manufacturing, Machine learning, Process monitoring, Multi-photon polymerization, Direct laser writing

## Abstract

This document describes the collection and organization of a dataset that consists of raw videos and extracted sub-images from video frames of a promising additive manufacturing technique called Two-Photon Lithography (TPL).  Four unprocessed videos were collected, with each video capturing the printing process of different combinations of 3D parts on different photoresists at varying light dosages.  These videos were further trimmed to obtain short clips that are organized by experimental parameters. Additionally, this dataset also contains a python script to reproduce an organized directory of cropped video frames extracted from the trimmed videos. These cropped frames focus on a region of interest around the parts being printed. We envision that the raw videos and cropped frames provided in this dataset will be used to train various computer vision and machine learning algorithms for applications such as object segmentation and localization applications. The cropped video frames were manually labelled by an expert to determine the quality of the printed part and for printing parameter optimization as presented in “Automated Detection of Part Quality during Two-Photon Lithography via Deep Learning” [1].

Specifications Table

**Table:** 

Subject	Artificial Intelligence
Specific subject area	Deep learning for part quality detection via image classification in two-photon lithography processes
Type of data	Videos Code
How data were acquired	Recorded videos of two-photon lithography process on commercial IP-DIP photoresist and a custom photoresist Instruments: Nanoscribe GT system, 63 × objective lens with a numerical aperture of 1.4 as described in “Automated Detection of Part Quality during Two-Photon Lithography via Deep Learning” [1].
Data format	Raw videos Trimmed videos Python script
Parameters for data collection	The recorded videos capture two-photon lithography additive manufacturing processes operating under a wide variety of parameters. The parameters that were varied are scanning speed, laser intensity, discretization period, photoresist material, write patterns and actual design of the printed part.
Description of data collection	The videos were collected using the in-situ camera of the Nanoscribe GT system
Data source location	Lawrence Livermore National Laboratory Livermore United States of America
Data accessibility	Public Repository: Mendeley Repository name: Two Photon Lithography Additive Manufacturing: Video Dataset of Parameter Sweep of Light Dosages, Photo-curable Resins, and Structures Data identification number: 10.17632/9skghw3xp4.1 Direct URL to data: https://data.mendeley.com/datasets/9skghw3xp4
Related research article	Authors: Xian Yeow Lee, Sourabh K. Saha, Soumik Sarkar, Brian Giera Title: Automated Detection of Part Quality During Two Photon Lithography via Deep Learning Journal: Additive Manufacturing DOI: https://doi.org/10.1016/j.addma.2020.101444

Value of the Data

This dataset contains videos capturing three universally encountered polymerization states of a two-photon lithography (TPL) process (uncured, cured and damaged) under a wide variety of operating conditions, which occur in any TPL system. While routinely collected, this data is expensive to obtain or impossible without access to this particular representative additive manufacturing system.
This dataset benefits researchers working in the field of additive manufacturing and also applied machine learning and computer vision algorithms, specifically for image processing and real-time detection.
The raw videos can be used to test new machine learning and computer vision algorithms for automating tasks such as segmentation, localization, and classification of TPL processes whether with supervised, unsupervised, or semi-supervised approaches.
The experimental parameters (laser intensity, write speed, discretization period) provided can be easily used in conjunction with the videos to identify ideal operating conditions to reproduce the defect-free parts.
This dataset can seed efforts for real-time process monitoring and, in some cases, rectification in addition to automated part detection for industrial scale production, e.g. in high throughput TPL system.
While the chemical mechanisms of polymerization are known, there are few, if any, generally applicable models that can be used *a priori* to predict the specific light dosage parameters that result in one of the three photo-polymerization states. The experimental design and collected data can be used to generate empirically derived data driven models for predicting photo polymerization outcomes.

## 1 Data description

### 1.1 Raw videos

**Fig. 1:**
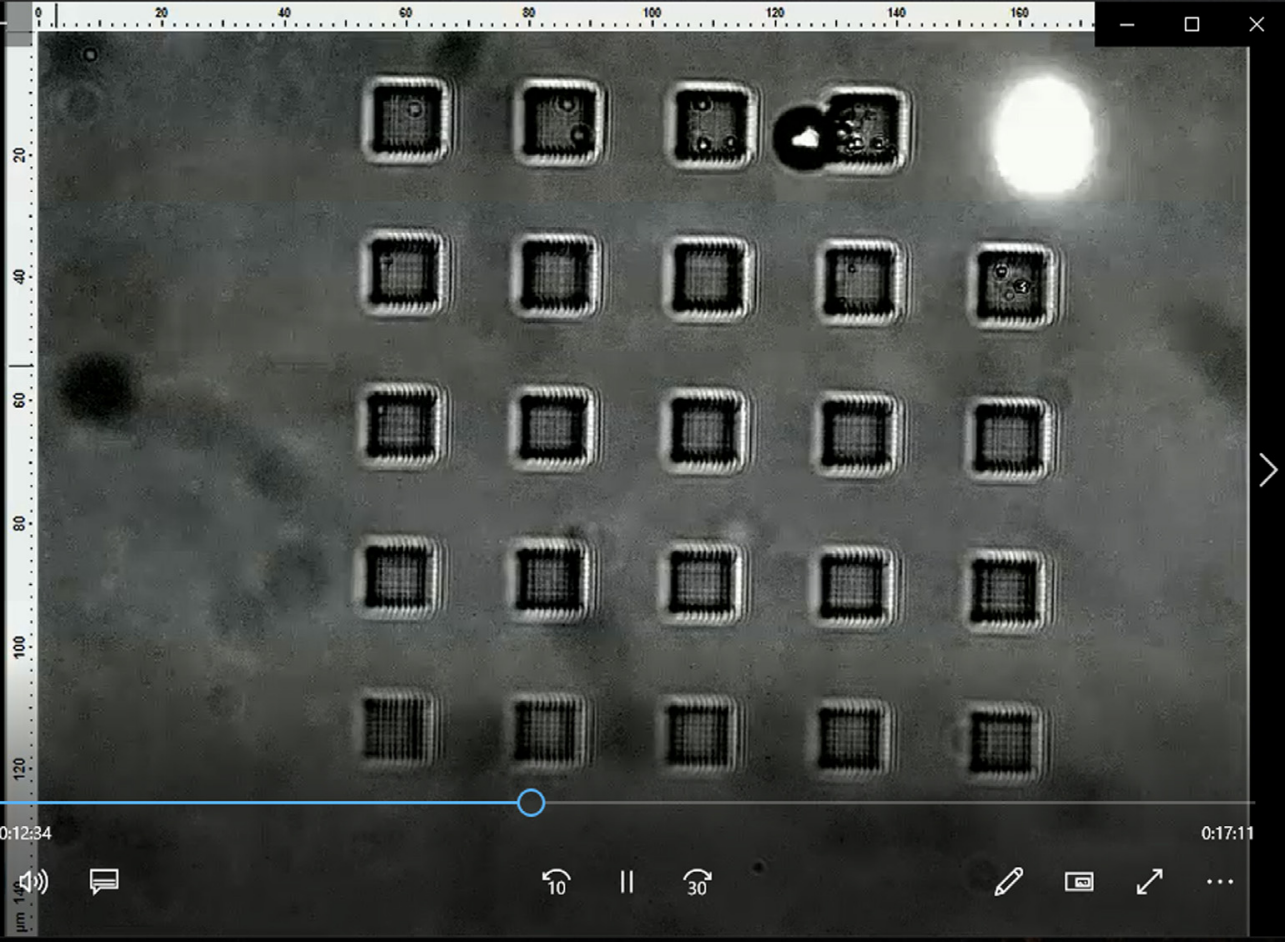
Example screenshot taken from the *Cuboid-Logpile-IPDIP.mp4* video.

The dataset contains four raw videos in .mp4 format. Each video captures of the printing process of a specific part design with a specific write pattern and photoresist. For every video, scale bars for the height and width are provided, in microns that indicate the distance/pixel resolution. In each video, only the discretization period, writing speed, and laser intensity of the TPL process were varied. Table 1 summarizes the content corresponding to each video file and Fig. 1 shows an example frame of the video extracted from “Cuboid-Logpile-IPDIP.mp4”. A detailed schematic of the experimental system used to collect the data is illustrated in Fig. 1 of the related research article [1]. Further details of the experimental design are elaborated in the next section.

**Table 1:** Summary of video file contents.

Video File Name	Summary
Cuboid-Logpile-IPDIP.mp4	Video of the printing process of 308 cuboid-shaped parts using a log-pile write pattern on the commercial IP-DIP photoresist.
Cuboid-Logpile-Custom.mp4	Video of the printing process of 325 cuboid-shaped parts using a log-pile write pattern on a custom photoresist.
Cones-Circular-IPDIP.mp4	Video of the printing process of 150 conical-shaped parts using a circular write pattern on the commercial IP-DIP photoresist.
Cones-Logpile-IPDIP.mp4	Video of the printing process of 260 conical-shaped parts using a log-pile write pattern on the commercial IP-DIP photoresist.

### 1.2 Trimmed videos

In addition to the raw videos, this dataset contains multiple short video clips that are derived from the raw videos above. In each of the raw videos above, there is a maximum of 25 printed parts in one scene before the printing stage is moved to a new scene. We refer to each scene as a quadrant and the relationship between each quadrant and printing parameters is explained in the next section. Each of the raw videos above contains multiple quadrants. For ease of further processing, we manually trimmed and created multiple shorter video clips such that each video clip is only limited to a single quadrant. These short clips are organized and stored into four folders with the names corresponding to the raw video file names as shown in Table 2.

**Table 2:** Summary of trimmed video clips derived from raw videos.

Raw Video	Folder Name	Number of Short Video Clips
Cuboid-Logpile-IPDIP.mp4	Cuboid-Logpile-IPDIP	14
Cuboid-Logpile-Custom.mp4	Cuboid-Logpile-Custom	12
Cones-Circular-IPDIP.mp4	Cones-Circular-IPDIP	6
Cones-Logpile-IPDIP.mp4	Cones-Logpile-IPDIP	11

## 2 Experimental design, materials, and methods

In this section, we describe the different experimental conditions that were used to collect the raw video data.

### 2.1 Experimental design for cuboid videos

The first and second videos listed in Table 1 show the printing process of cuboid structures described here [2]. Each cuboid structure has the dimension of 10 µm × 10 µm × 10 µm with a Z-layer spacing of 0.6 µm. The cuboids are discretized in the X-Y plane in the form of a Cartesian grid log-pile. The discretization period of this grid is varied across different test runs. Both videos have a total of four test runs.

Each test run comprises of printing the cuboid parts at a fixed writing speed and discretization period but at varying laser power levels. In each test run, the laser power levels range from 1% to 100% of maximum laser intensity, which would yield a total of 100 printed cuboids for each test run. That said, there are a few test runs that have less than 100 cuboids. This occurs when the printed cuboids are consistently damaged, and it was clear that the laser intensity was beyond the ideal operating conditions; hence the test run was ended prematurely.

In the videos, each test run is split into four quadrants comprising of 25 printed cuboids per quadrant. Within each quadrant, the printed cuboids are separated 25 µm from each other along the row and column axes, yielding a total of five rows and five columns in each quadrant. The rows are numbered from the bottom up, and columns are numbered from left to right. The first print always occurs in the first row and first column, followed by the second print in the first row and second column. The printing conditions in the first quadrant has laser power levels starting from 1% up to 25%, the second quadrant from 26% to 50%, the third quadrant from 51% to 75% and the fourth quadrant from 76% up to 100%. A summary of the experimental parameters of each test run alongside the time point in the videos, which coincides with the change in parameters, is tabulated in Table 3 for the Cuboid-Logpile-IPDIP video and Table 4 for the Cuboid-Logpile-Custom video.

**Table 3:** Test conditions for Cuboid-Logpile-IPDIP video

Test run	Number of quadrants	Video time location (min)	Discretization period (µm)	Speed (mm/s)	Power ramp (% of 50 mW)
1	2 and a partial 3rd	0:0–7:33	0.5	1	1–57
2	3 and a partial 4th	7:44–15:27	1	1	1–76
3	3	15:28–21:53	2.5	1	1–75
4	4	21:54 to end	1	10	1–100

**Table 4:** Test conditions for Cuboid-Logpile-Custom video.

Test run	Number of quadrants	Video time location (min)	Discretization period (µm)	Speed (mm/s)	Power ramp (% of 50 mW)
1	3	0:0–6:39	0.5	1	1–75
2	3	6:40–12:41	1	5	1–75
3	3	12:42–18:34	1	10	1–75
4	4	18:35 to end	2.5	10	1–100

**Fig. 2:**
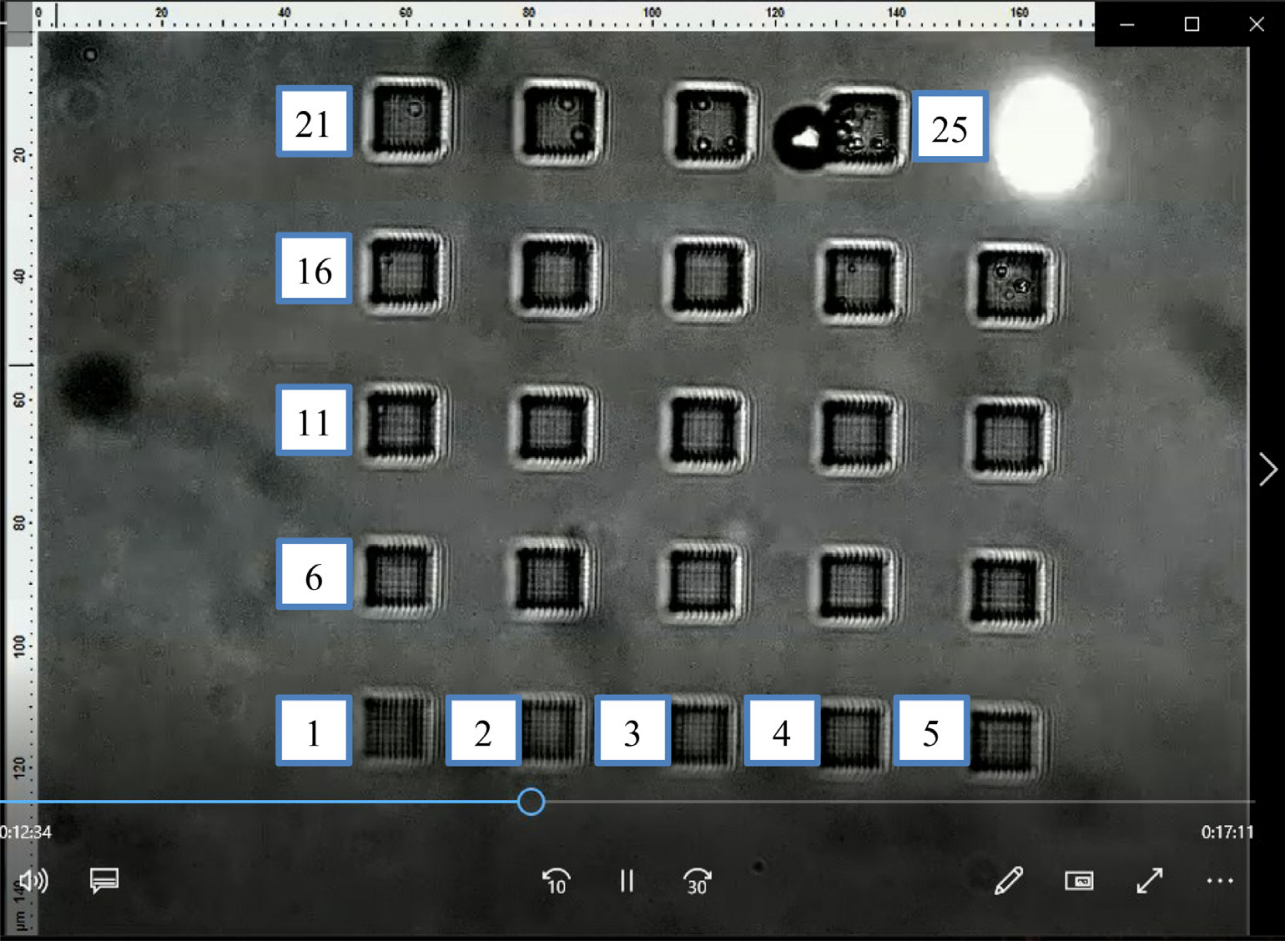
Illustration of numbering convention of printed parts for laser power increments. In one quadrant, the laser power level increases from 1% to 25%. In the next quadrant within the same test run, the first printed part begins with a laser power level of 26–50%. After a single test run is completed, the next test run begins with laser power of 1% again, but at a different discretization period and writing speed.

Fig. 2 illustrates an example of the numbering convention of the printed cuboids in a single quadrant. Each quadrant of a test run (i.e., a set of 25 prints) is located within the field-of-view of a single frame. While the printing is occurring in the same quadrant, the stage is held stationary in the X-Y plane (but not in Z plane). The X-Y stage is moved to a different location after the 25th cuboid in the quadrant is printed.

### 2.3 Experimental design for cone videos

The third and fourth videos listed in Table 1 consists of conical structures. The numbering convention of the printed cones and experimental design, i.e. varying discretization period and writing speed, is the same as described above. The laser power levels range from 1% to 100% in each test run, and each test run is also divided into four quadrants, with some test runs having less than 100 printed parts.

The designs of the cones in the videos are truncated cones with a base diameter of 10 µm, height 7 µm, and 6.7 µm top diameter. The cones are sliced at a Z-layer spacing of 0.6 µm. In the “Cones-Circular-IPDIP.mp4” video, each layer of the cone's cross-section is discretized in terms of 8 concentric rings from the exterior boundary and spaced at a uniform radial spacing. This spacing is varied across the prints. The remainder of the central region in each cone is discretized as a Cartesian log-pile grid with a spacing of 0.5 µm. The grid spacing of this central region is not varied across the prints. In the “Cones-Logpile-IPDIP.mp4” video, each layer of the cones is discretized in the X-Y plane in the form of a cartesian grid log-pile with a spacing of 0.5 µm. Tables 5 and 6 summarize the experimental parameters used in the printing process captured by both videos.

**Table 5:** Test conditions for Cones-Circular-IPDIP video.

Test run	Number of quadrants	Video time location (min)	Discretization period (µm)	Speed (mm/s)	Power ramp (% of 50 mW)
1	2	0:0–5:30	0.2	1	1–50
2	2	5:31–10:12	0.4	1	1–50
3	2	10:13 to end	0.4	10	1–50

**Table 6:** Test conditions for Cones-Logpile-IPDIP video.

Test run	Number of quadrants	Video time location (min)	Discretization period (µm)	Speed (mm/s)	Power ramp (% of 50 mW)
1	2	0:0–5:48	0.2	1	1–50
2	2	5:49–10:11	0.4	1	1–50
3	3	10:12–15:29	0.6	1	1–60
4	4	15:30 to end	0.4	10	1–100

### 2.4 Extraction of video frames

In this section, we describe the method used to extract cropped video frames focusing on a single 3D part from the raw videos. For the ease of organization, the raw videos were manually split into multiple shorter clips where each video clip only depicts the printing process of one quadrant as listed in Table 2.

For each of these quadrant clip, we empirically determine the specific pixel location of the first part that was being printed and extracted a 110 × 110 pixel^2^ region of interest around the part. Since all the printed parts are equally spaced apart within a quadrant, the pixel coordinates of the region of interest of the rest of the parts can be computationally determined based on the pixel location of the first part.

A python script was used to read the video clips using OpenCV [3]. Based on the pixel location of the first print, the script determines the location of the rest of the parts and crops out the region of interest from every frame of the video and saves it into an organized directory. The python script, named *preprocess.py,* is provided together in the attached dataset.

## Declaration of Competing Interest

The authors declare that they have no known competing financial interests or personal relationships which have, or could be perceived to have, influenced the work reported in this article.
